# Commentary: LRP1 Is a Master Regulator of Tau Uptake and Spread

**DOI:** 10.3389/fneur.2020.557509

**Published:** 2020-12-23

**Authors:** Conor Fearon, Tim Lynch

**Affiliations:** ^1^Centre for Brain Health, Dublin Neurological Institute at the Mater Misericordiae University Hospital, Dublin, Ireland; ^2^Department of Neuropathology, Beaumont Hospital, Dublin, Ireland; ^3^Health Affairs, University College Dublin, Dublin, Ireland

**Keywords:** tau propagation, LRP1, tauopathies, Alzheimer's disease, amyloid (A) 42, corticobasal degeneration (CBD), progressive supranuclear palsy, frontotemporal lobar degeneration

Although present as a physiological intracellular protein, pathological aggregation of microtubule-associated tau plays a central role in the pathophysiology of neurodegenerative disease including progressive supranuclear palsy (PSP), corticobasal degeneration (CBD), frontotemporal dementia (FTD), chronic traumatic encephalopathy (CTE), and Alzheimer's disease (AD)—the tauopathies (1). Remarkably, autopsy of patients with tauopathies shows spared gyri often adjacent to devastated gyri (2). Moreover, the topography of neurodegeneration often dictates the clinical syndrome. Why are some brain regions devastated and others spared in the tauopathies? Perhaps abnormal protein spreads within certain pathways but spares others. Like other proteinopathies, animal, and cell models demonstrate spreading of the pathogenic protein from cell to cell in a prion-like fashion. The mechanism of interneuronal spread of tau is poorly understood.

In their recent study, Rauch and colleagues showed that neuronal surface low-density-lipoprotein-receptor-related protein-1 (LRP1), mediates internalization of both physiological tau and pathogenic tau oligomers, and hence, their subsequent neuronal spread (3). The investigators demonstrate that *LRP1* loss reduces internalization of tau in cell culture. This interaction occurs via lysine residues within the tau microtubule-binding region (MTBR). Chemical blockade of these residues prevents tau endocytosis. The authors then used a murine model of tau spread that discriminates between transduced neurons expressing human tau (hTau) and neurons that receive hTau through spread. They demonstrated that downregulation of *LRP1* and subsequent expression of mutant tau limits the spread of tau compared to *LRP1*-wild-type mice.

There are no disease-modifying treatments for the tauopathies, although there are currently 92 clinical trials ongoing (4). Several approaches targeting physiological and pathological tau have been tried, including inhibition of tau aggregation, active and passive immunotherapies and tau silencing with antisense oligonucleotides (5). Many target either monomeric, aggregated, phosphorylated, or conformationally altered forms of tau (5). The discovery that LRP1 is a gatekeeper for transmission of both physiological *and* pathological tau is highly relevant for these trials. There are, however, some caveats: whether pathogenic tau spread is associated with subsequent neurodegeneration was not assessed; effects of fibril conformation, selective vulnerability of specific brain regions, tau mutations and background genomics, other pathogenic proteins, immune processes, and glial pathology may all complicate the neurodegenerative picture in humans compared with the otherwise healthy mouse brains used by Rauch et al. (3).

Furthermore, it remains unclear why neurodegeneration occurs in tauopathies associated with an abnormal ratio of 3-repeat (3R) to 4-repeat (4R) tau (either an excess of 3R or an excess of 4R tau). Since the LRP1-interacting tau residues are located within the MTBR, the ratio 3R to 4R tau *in vivo* may be highly relevant to LRP1's pathophysiological role. Although there were no differences in tau uptake between the six main tau isoforms, much of Rauch and colleagues' ligand-binding data refers to 4R tau (3). There is growing evidence, however, of a much wider range of specific tau conformational structures among the tauopathies (6). If LRP1 binds and transmits specific tau conformations differently, these findings may have greater relevance for specific tauopathies where post-translation modifications or other factors impact conformation.

Interestingly, *astrocytic* LRP1 clears Aβ_42_ amyloid across the blood brain barrier (BBB), reducing its cortical deposition (7). Whether LRP1 also clears tau across the BBB was not studied. Although tau deposition probably occurs later than Aβ_42_ amyloid in AD, its distribution correlates more closely with the clinical syndrome. Conversely, Aβ_42_ amyloid deposition is not a prominent feature in the primary tauopathies (such as PSP, CBD and FTD). Rauch and colleagues study was limited to LRP1-mediated *neuronal* spread of tau. Given that many tauopathies have significant glial pathology, the effect of LRP1 on BBB clearance and transmission of tau in other cells types must be investigated.

However, an amyloid precursor protein (APP) mouse model demonstrated that LRP1 enhances production of Aβ_42_ amyloid via APP processing and that this effect outweighs BBB clearance of Aβ_42_ amyloid (8). This dual role of LRP1 in AD pathophysiology is therefore complex and can be modulated by other proteins. The presence of Aβ_42_ amyloid increases ectodomain shedding of the low density lipoprotein receptor and LRP1 in human brain endothelial cells, reducing Aβ_42_ amyloid clearance across the BBB (9). The effect of Aβ_42_ amyloid on LRP1-related APP processing or LRP1-related tau transmission has not been studied, but tau transmission may be similarly reduced by LRP1 shedding. Apolipoprotein E (APOE) also binds LRP1 and Rauch and colleagues demonstrated that tau competes with APOE isoforms for LRP1 binding (3). When Aβ_42_ amyloid is present with different APOE isoforms (APOE ε2, APOE ε3, and APOE ε4), LRP1 shedding is isoform-dependent with greater LRP1 shedding occurring in the presence of APOE ε4 and lesser shedding occurring in the presence of APOE ε2 (9). The possession of the APO ε4 allele is the strongest genetic risk factor for late-onset AD (10). However, the APOE ε2 allele is associated with increased tau pathology in mouse models and in brains of patients with PSP and CBD (11). Thus, APOE ε4 may increase Aβ_42_ amyloid cortical accumulation (subsequent tau spread may be through alternative mechanisms) while APOE ε2 may facilitate clearance of Aβ_42_ amyloid and spreading of tau in the primary tauopathies. The effect of APOE on LRP-1-related APP processing has not been studied. Although APOE competes with tau for LRP1, Rauch and colleagues did not show a difference in neuronal tau transmission between APOE isoforms *in vitro*. The effects of APOE isoforms on tau transmission in astrocytic or endothelial cells and *in vivo*, however, may be a more appropriate comparison. Multiple factors including APOE isoforms, tau conformational strains and the epichaperone system might therefore modulate tau transmission via LRP1, partially explaining the diverse neuropathologies and clinical syndromes found in different tauopathies. The distribution of LRP1 in vulnerable brain regions in specific tauopathies would be of great interest in this respect.

Rauch and colleagues provide new insight into tau physiology and potential therapeutic targets for tauopathies: LRP1; the associated lysine residues within the MTBR; and the yet unstudied processes downstream of this LRP1-tau interaction. Given the complex modulating effect of other proteins on LRP1 transmission, a more direct target may be the MTBR lysine residues. Indeed, antibodies directed against the MTBR (E2814 and DC8E8) show promise in preclinical studies by reducing tau internalization (4). However, *in vitro* and animal data do not always translate to human studies. With numerous failed clinical trials, no community understands this greater than the neurodegenerative fraternity. Success of any future treatment will not rely solely on reduced tau transmission, but on reduced neurodegeneration and motor/cognitive impairment in these relentlessly progressive conditions.

## Author Contributions

CF: manuscript conception, organization, and writing of first draft on manuscript. TL: manuscript conception and review of manuscript. All authors contributed to the article and approved the submitted version.

## Conflict of Interest

The authors declare that the research was conducted in the absence of any commercial or financial relationships that could be construed as a potential conflict of interest.
